# Integrated RNA and metabolite profiling of urine liquid biopsies for prostate cancer biomarker discovery

**DOI:** 10.1038/s41598-020-60616-z

**Published:** 2020-02-28

**Authors:** Bongyong Lee, Iqbal Mahmud, John Marchica, Paweł Dereziński, Feng Qi, Fubo Wang, Piyush Joshi, Felipe Valerio, Inoel Rivera, Vipul Patel, Christian P. Pavlovich, Timothy. J. Garrett, Gary P. Schroth, Yinghao Sun, Ranjan J. Perera

**Affiliations:** 1 Cancer and Blood Disorders Institute, Johns Hopkins All Children’s Hospital, 600 6th Avenue South, St. Petersburg, FL 33701 USA; 2 Department of Oncology, Sydney Kimmel Cancer Center, Johns Hopkins University School of Medicine, 401 N. Broadway, Baltimore, MD 21287 USA; 3 The James Buchanan Brady Urological Institute, Department of Urology, The Johns Hopkins University School of Medicine, 4940 Eastern Avenue, Baltimore, MD 21224 USA; 4 Sanford Burnham Prebys Medical Discovery Institute, 10901 N Torrey Pines Rd, La Jolla, CA 92037 USA; 5 Department Pathology, Immunology and Laboratory Medicine, University of Florida, College of Medicine, 1395 Center Drive, Gainesville, FL 32610 USA; 6 Department of Inorganic and Analytical Chemistry, Poznan University of Medical Sciences, Grunwaldzka 6 Street, 60-780 Poznan, Poland; 7 Department of Urology, Shanghai Changhai Hospital, Second Military Medical University, 168 Changhai Road, Shanghai, China; 8 Florida Urology Associates, 1812 N. Mills Avenue, Orlando, FL 32803 USA; 9 Global Robotics Institute, 410 Celebration Place, Suite 200, Celebration, FL 34747 USA; 10 Illumina, Inc., 5200 Illumina Way, San Diego, CA 92122 USA

**Keywords:** Tumour biomarkers, Prostate cancer

## Abstract

Sensitive and specific diagnostic and prognostic biomarkers for prostate cancer (PCa) are urgently needed. Urine samples are a non-invasive means to obtain abundant and readily accessible “liquid biopsies”. Herein we used urine liquid biopsies to identify and characterize a novel group of urine-enriched RNAs and metabolites in patients with PCa and normal individuals with or without benign prostatic disease. Differentially expressed RNAs were identified in urine samples by deep sequencing and metabolites in urine were measured by mass spectrometry. mRNA and metabolite profiles were distinct in patients with benign and malignant disease. Integrated analysis of urinary gene expression and metabolite signatures unveiled an aberrant glutamate metabolism and tricarboxylic acid (TCA) cycle node in prostate cancer-derived cells. Functional validation supported a role for glutamate metabolism and glutamate oxaloacetate transaminase 1 (GOT1)*-*dependent redox balance in PCa, which could be exploited for novel biomarkers and therapies. In this study, we discovered cancer-specific changes in urinary RNAs and metabolites, paving the way for the development of sensitive and specific urinary PCa diagnostic biomarkers either alone or in combination. Our methodology was based on single void urine samples (i.e., without prostatic massage). The integrated analysis of metabolomic and transcriptomic data from these liquid biopsies revealed a glutamate metabolism and tricarboxylic acid cycle node that was specific to prostate-derived cancer cells and cancer-specific metabolic changes in urine.

## Introduction

More than 180,000 men are diagnosed with prostate cancer (PCa) in U.S. in the 2016, where 26,000 of these patients will die of the disease^1^. PCa is one of the second most frequently diagnosed cancer deaths among men worldwide^2^. The radiotherapy and surgery for localized PCa are known to be effective, however the prognosis for patients with the progressive disease is poor. A test to detect PCa with high sensitivity and specificity at an early stage is a clinical imperative. Moreover, there is a vital need for novel therapeutic tactics to manage this insidious and prevalent disease.

Serum prostate specific antigen (PSA) levels are been used for PCa diagnosis and screening for over thirty years, and digital rectal examination (DRE) for even longer^3^. However, PSA has modest sensitivity and specificity and does not discriminate indolent from aggressive cancers^3,4^. Prostate cancer antigen 3 (PCA3), a prostate-specific non-coding RNA, was approved by the FDA in 2012 as the first PCa molecular diagnostic test for a particular clinical indication (need for recurrence prostate biopsies in men aged >50 years with assumed PSA levels and/or DRE and/or one or more earlier negative biopsies)^5^. Nevertheless, the importance of the PCA3 test is limited by substantial individual variability, better performance in the replication biopsy setting, and contradictory data on the relationship between score and cancer grade using the most common edge of 35^6^. Hence, there is a dismal need for novel molecular diagnostic tools to more precisely detect and predict the performance of localized PCa.

The kidneys produce urine to eliminate soluble waste from the bloodstream. Urine is an abundant biofluid for molecular or cellular analyses and is useful in the diagnosis and management of bladder, ovarian, and kidney diseases^7–9^. Urine contains over 2500 metabolites^10^ and provides a window through which to view cellular biochemical reactions and intermediary metabolism. The metabolite signature in urine will reflect the impact of gene regulation, enzyme activities, and alterations in metabolic reactions occurring in the different cell types found along the urogenital tract.

Cancer cells exhibit perturbed metabolism that enables proliferation and survival^11^. Therefore, metabolomic profiling has been a fruitful approach for the identification of early cancer biomarkers^12,13^. Furthermore, certain metabolic states are associated with prognosis in advanced cancers^13^. Several metabolomics studies have revealed PCa-specific metabolic phenotypes in serum, tissue, and urine^14,15^. Indeed, an intermediate metabolite of glycine synthesis and degradation, sarcosine, has been described as a putative PCa biomarker in urine^16^. Cancer cells exhibit perturbed metabolism that enables proliferation and survival^11^. Therefore, metabolomic profiling has been a fruitful approach for the identification of early cancer biomarkers^12,13^. Furthermore, certain metabolic states are associated with prognosis in advanced cancers^13^. Several metabolomics studies have revealed PCa-specific metabolic phenotypes in serum, tissue, and urine^14,15^. Indeed, an intermediate metabolite of glycine synthesis and degradation, sarcosine, has been described as a putative PCa biomarker in urine^16^. However, the utility of sarcosine as a biomarker is provocative and clinical validation has been indefinable^17^.

Metabolomics data have been integrated with comprehensive gene expression analyses to better interrogate complex gene and metabolic networks. Integrating multiple aspects of biological complexity using different unsupervised approaches can help to pinpoint the most important and reproducible pathways driving biological processes and hence reveal robust biomarkers or promising drug targets^18,19^. Here we performed metabolite profiling and high-throughput RNA sequencing obtained urine from benign prostatic hyperplasia, prostatitis, and PCa patients. Our aims were to (a) discover cancer-specific changes in the urine with utility as sensitive and specific PCa biomarkers either alone or in combination and (b) identify novel drug targets for PCa. Importantly, our approach used single void urine samples (i.e., without prostatic massage) as proof-of-principle of how a simple urine specimen can be used for biomarker and target discovery. Integrated analysis of metabolomic and transcriptomic data from these liquid biopsies revealed a glutamate metabolism and tricarboxylic acid cycle node that was specific to prostate-derived cancer cells and cancer-specific metabolic changes in urine. Functional validation *in vitro* provided mechanistic support for a pivotal role for GOT1-dependent glutamate metabolism in redox balance and cancer progression.

## Results

### Deep sequencing of urine-secreted mRNAs

Normal voided urine from men contains small numbers of exfoliated cells from different parts of the urinary tract including urothelial cells, squamous cells, renal tubular cells, and glandular cells including prostate epithelial cells^20^. PCa cells are shed into urine and can be successfully isolated, processed, and analyzed by various molecular techniques^3^, thereby providing a rich substrate for biomarker detection. We sought to exploit this readily accessible and copious substrate from PCa patients for biomarker discovery and, in turn, elucidate novel mechanistic aspects of PCa.

Quality output from current next-generation sequencing (NGS) technology depends on the availability of high-quality RNA. An initial challenge was that the quality and quantity of RNA extracted from the very small number of exfoliated cells in urine was poor^21^ (Supplementary Fig. 1). To overcome the problem, we performed sequence-specific capture (Illumina TruSeq RNA Access) with the urine samples to reduce ribosomal RNA and enrich for exonic RNA sequences. With this approach, we successfully sequenced 11 PCa (for clinical details, see Supplementary Table 1), 12 normal, and one pooled set of three normal samples (combined due to individually low RNA yields). The 3825 RNA transcripts that were detected in 20 samples readily but not perfectly segregated into normal and PCa groups (Supplementary Fig. 2). We concluded that RNA expression analysis of urine liquid biopsies by itself was unlikely to reveal sensitive and specific PCa biomarkers.

We next identified cancer-specific gene signatures. Among 5510 differentially expressed transcripts, 4662 had RPKM values greater than one, and 116 transcripts (110 genes) were significantly up- or downregulated in PCa (Table 1). Known PCa markers were upregulated in PCa urine (Supplementary Table 2), and differentially expressed genes were enriched for a number of important cancer pathways including PCa signaling, molecular mechanisms of cancer, PI3K/AKT signaling, and NF-κB signaling (Supplementary Table 3). To our knowledge, this is the first time that RNA-seq has been successfully applied to urine samples to profile coding genes.

**Table 1:** Differentially expressed genes in prostate cancer urine samples.

Gene	Wilcoxon p-value	log2 fold-change	Gene	Wilcoxon p-value	log2 fold-change	Gene	Wilcoxon p-value	log2 fold-change
	Upregulated			Downregulated			Downregulated	
*ACE*	0.04	1.0	*ACSL3*	0.02	−1.3	*LMO7*	0.00	−3.0
*ATF1*	0.01	0.6	*ADM*	0.04	−2.5	*LRMP*	0.02	−3.2
*BRD3*	0.03	0.2	*BAZ2A*	0.01	−0.8	*MALAT1*	0.00	−3.2
*CCNB1IP1*	0.01	0.4	*BCL10*	0.00	−2.3	*MCL1*	0.03	−4.0
*CDC14A*	0.03	0.3	*BIRC3*	0.04	−4.1	*MDM2*	0.03	−1.2
*CDK8*	0.01	0.2	*BRWD3*	0.00	−1.5	*MYD88*	0.02	−4.2
*ELK4*	0.02	0.9	*CDKL5*	0.01	−1.1	*NCSTN*	0.02	−1.7
*EPCAM*	0.04	0.4	*CDKN1B*	0.02	−1.4	*NDE1*	0.02	−2.0
*FH*	0.04	0.0	*CHIC2*	0.01	−1.8	*NIPBL*	0.02	−1.3
*GMPS*	0.01	0.2	*CIC*	0.02	−3.9	*NOTCH1*	0.01	−2.9
*GNAS*	0.02	0.8	*CREBBP*	0.03	−1.2	*NT5C2*	0.02	−2.0
*GOT1*	0.02	0.5	*DDB2*	0.03	−1.3	*NUP98*	0.01	−2.3
*GRHPR*	0.00	1.3	*DOT1L*	0.01	−1.2	*PER1*	0.00	−1.9
*HDAC6*	0.04	0.6	*ELF4*	0.02	−1.8	*PICALM*	0.01	−3.0
*HSP90AB1*	0.04	0.6	*EP300*	0.01	−0.8	*PLCG2*	0.03	−1.6
*LRPPRC*	0.02	0.7	*EPHA2*	0.00	−3.2	*PRDM1*	0.00	−2.6
*MSH3*	0.00	0.1	*ERBB3*	0.04	−0.6	*PRSS8*	0.00	−3.3
*NACA*	0.03	1.1	*ERCC5*	0.03	−0.9	*PTK2B*	0.00	−2.1
*NPM1*	0.01	2.8	*ETV6*	0.04	−1.2	*RAF1*	0.04	−1.6
*PFDN5*	0.02	0.7	*FLCN*	0.00	−1.0	*RCOR1*	0.00	−1.3
*PHB*	0.01	0.3	*FOSB*	0.03	−2.1	*RNF213*	0.03	−1.5
*PHF6*	0.04	0.2	*GIT2*	0.04	−5.1	*SH3BP1*	0.01	−1.6
*PIK3R1*	0.00	1.4	*GNAQ*	0.02	−1.0	*SMAD3*	0.02	−1.4
*PTK2*	0.03	1.1	*HDAC4*	0.02	−1.3	*STRN*	0.02	−0.3
*PTPN2*	0.02	2.8	*HIPK1*	0.03	−1.6	*TMEM127*	0.02	−1.9
*RPL22*	0.01	0.9	*HIST1H2BO*	0.04	−0.9	*TMPRSS2*	0.02	−2.0
*RPS11*	0.01	0.9	*ICAM1*	0.03	−3.1	*TNFAIP3*	0.03	−2.2
*SDHC*	0.02	2.9	*IGF1R*	0.04	−1.1	*TOP1*	0.02	−1.3
*SDHD*	0.03	1.0	*IRF1*	0.03	−2.1	*TPM4*	0.02	−2.2
*SMAD4*	0.02	0.0	*JAK2*	0.04	−0.8	*TYK2*	0.02	−1.9
*SMARCB1*	0.00	0.3	*KDM5A*	0.04	−0.1	*VHL*	0.04	−1.8
*TCEA1*	0.03	0.1	*KDM5C*	0.01	−1.3	*WDFY3*	0.04	−1.0
*TERF2*	0.02	0.3	*KIAA0232*	0.01	−1.0	*WDR1*	0.04	−1.7
*TFG*	0.03	0.1	*KMT2B*	0.03	−0.7	*WSB1*	0.03	−1.7
*TMEM230*	0.02	0.2	*KMT2C*	0.03	−0.9	*XIAP*	0.00	−1.4
*ZMYND11*	0.00	3.1	*KMT2D*	0.02	−0.9	*ZNF217*	0.00	−1.1
*ZNF585B*	0.02	0.4	*LAMP2*	0.02	−2.9			

**Figure 1:**
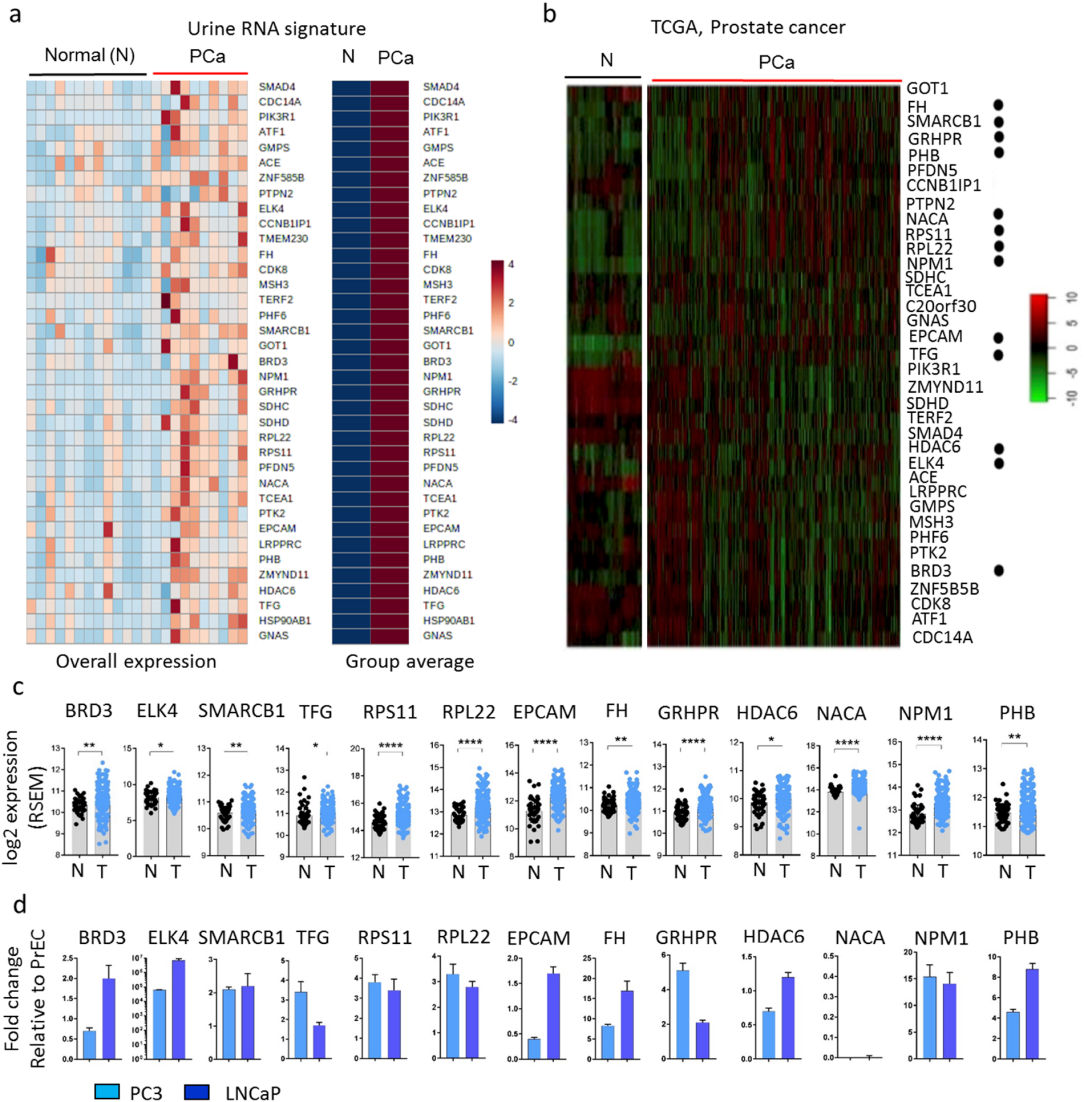
RNA-seq of cells extracted from the urine of patients with (n = 8) and without (n = 12) prostate cancer and validation of their differential expression in The Cancer Genome Atlas (TCGA) data. (**a)** A heatmap showing expression of 37 significantly upregulated genes in PCa cancer liquid biopsies. **(b,c)** Among 37 upregulated genes, 13 genes (*FH, SMARCB1, GRHPR, PHB, NACA, RPS11, RPL22, NPM1, EPCAM, TFG, HDAC6, ELK4, BRD3*) were significantly upregulated in primary tumors (n = 497) compared to normal (n = 52) in TCGA data. In the heatmap, black dots next to the gene name mark the genes upregulated in primary tumors compare to normal in TCGA data. The TCGA project for PCa data is publicly available for download at https://portal.gdc.cancer.gov/projects/TCGA-PRAD. **(d)** These 13 genes were also tested in two prostate cancer cell lines (LNCaP and PC3), and most were overexpressed apart from *NACA*, which was downregulated in both cell lines; *BRD3* and *EPCAM*, which were decreased in PC3 cells; and *HDAC6*, which was downregulated in LNCaP cells.

**Figure 2:**
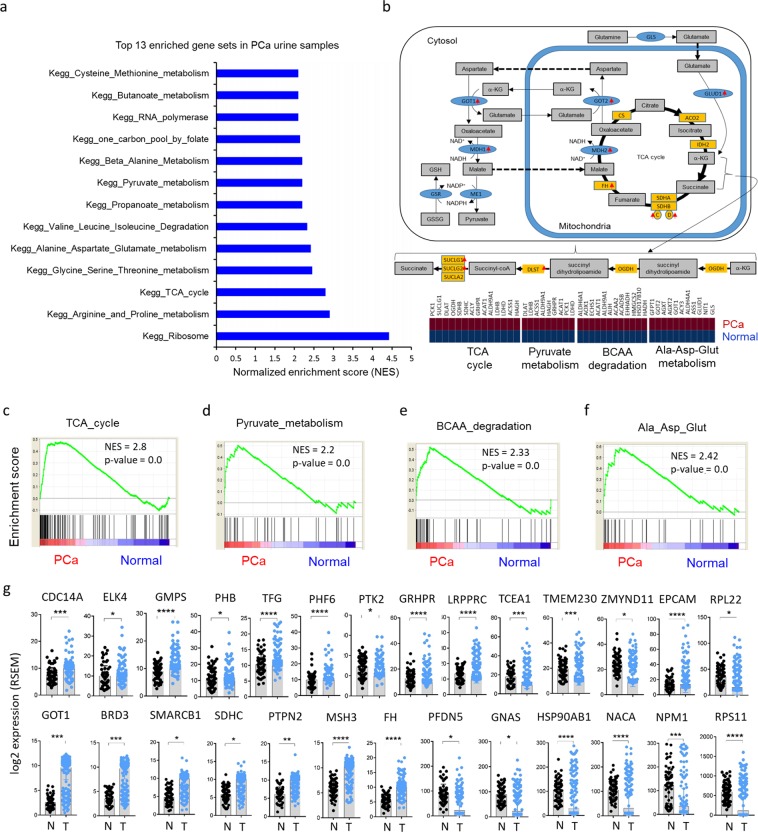
Gene set enrichment analysis (GSEA) of urine transcriptomes and validation of the expression of 37 genes in prostate tumor tissues. (**a)** Normalized Enrichment Score plot of the top 13 pathways in PCa urine. **(b)** Schematic view of TCA cycle and glutamate metabolism. Red arrows indicate upregulated genes in PCa urine compared to normal urine samples. **(c–f)** GSEA Plot for the TCA cycle, pyruvate metabolism, BCAA degradation, and alanine, aspartate, and glutamate metabolism, respectively. GSEA was conducted using GSEA software from the Broad Institute (http://software.broadinstitute.org/gsea/index.jsp). **(g)** The 37 gene signature from PCa patient urine RNA was confirmed in 65 pairs of tumors and adjacent normal tissue by RNA-seq data. Thirty four out of 37 genes were upregulated in PCa tumor tissue. Among them, 27 genes were significantly upregulated (Student *t*-test, *p < 0.05, **p < 0.01, ***p < 0.001, ****p < 0.0001).

Thirty-seven genes were significantly upregulated in PCa urine samples (Table 1 and Fig. 1a) compared to normal urine samples. To bolster confidence that the RNA originated from the patient’s cancer rather than contaminating cells, we examined their expression in The Cancer Genome Atlas (TCGA) data (Fig. 1b,c and Supplementary Table 4). Of these 37 genes, 35% (13/37) were significantly upregulated in primary tumors compared to normal (Fig. 1b,c). Three of these genes were transcription factors (*ELK4*, *SMARCB1*, *BRD3*) and six were known oncogenes (*TFG*, *NACA*, *BRD3*, *ELK4*, *NPM1*, *RPL22*)^22^. When quantified in two representative PCa cell lines (LNCaP and PC3), most transcripts were upregulated in both cell lines compared to normal prostate epithelial cells (PrEC) except for *NACA* (downregulated in both cell lines), *BRD3* and *EPCAM* (decreased in PC3 cells), and *HDAC6* (downregulated in LNCaP cells) (Fig. 1d). Gene set enrichment analysis (GSEA) revealed thirteen overrepresented pathways in PCa urine compared to normal urine samples based on normalized gene set enrichment scores (NES) (Fig. 2a). Among them, pyruvate metabolism, TCA cycle, branched chain amino acid (BCAA) degradation pathway, and alanine, aspartate, and glutamate metabolism were significantly enriched (Fig. 2b–f). It is well established that tumors predominantly reprogram metabolism in demand to satisfy three major functions: bioenergetics (central carbon metabolism), biosynthesis (the production of biomaterials such as nucleotides, amino acids, and lipids), and redox balance (chemistry of oxidation-reduction homeostasis status in metabolism)^23,24^. Together, these processes support cancer cell maintenance, proliferation, tumor initiation, growth, and progression and are considered central cancer metabolism dogma that allows cancer cells to adapt to changing cellular and physiological conditions in the context of growth-related signaling and stress. Key carbon metabolism pathways such as pyruvate metabolism and the TCA cycle, which are the source of energy; different amino acids pathways critical for the production of macromolecules such as protein, nucleotides, and lipids; and alanine, aspartate, and glutamate metabolism, which are essential for redox balance, were significantly upregulated in urine secreted PCa cells (Fig. 2b). These urinary cell transcriptional profiles are highly consistent with the principle of altered cancer metabolism and current knowledge of prostate cancer disease progression as determined in tissues and other biofluids^16,25^. Taken together, our data suggest that the transcriptional profiles generated from cells residing in urine from PCa patients are likely to originate from cancerous prostate epithelial cells rather than other urinary tract contaminants.

### Validation of urine gene signatures in tumor tissue

Since exfoliated cells in urine represent a mixture of cell types, we next established that the gene expression profiles of urine-exfoliated cells represented expression in prostate tissue using RNA-seq data derived from 65 PCa and matched normal prostate tissues^25^. Most of the 110 up- or downregulated genes in urine exfoliated cells (Table 1) agreed with tissue gene expression (Fig. 2g and Supplementary Table 5). Of 37 upregulated genes, 34 genes were upregulated in PCa tissue, and 27 of 34 genes (79%) were significantly upregulated (Fig. 2g). For the downregulated genes, 46 out of 73 genes (60%) were downregulated in PCa tissue compared to normal tissue (Supplementary Table 5). These results suggest that the PCa gene signature detected in urine exfoliated cells represents a *bone fide* PCa signature, especially with regard to upregulated genes, which may therefore represent more robust biomarkers.

**Figure 3:**
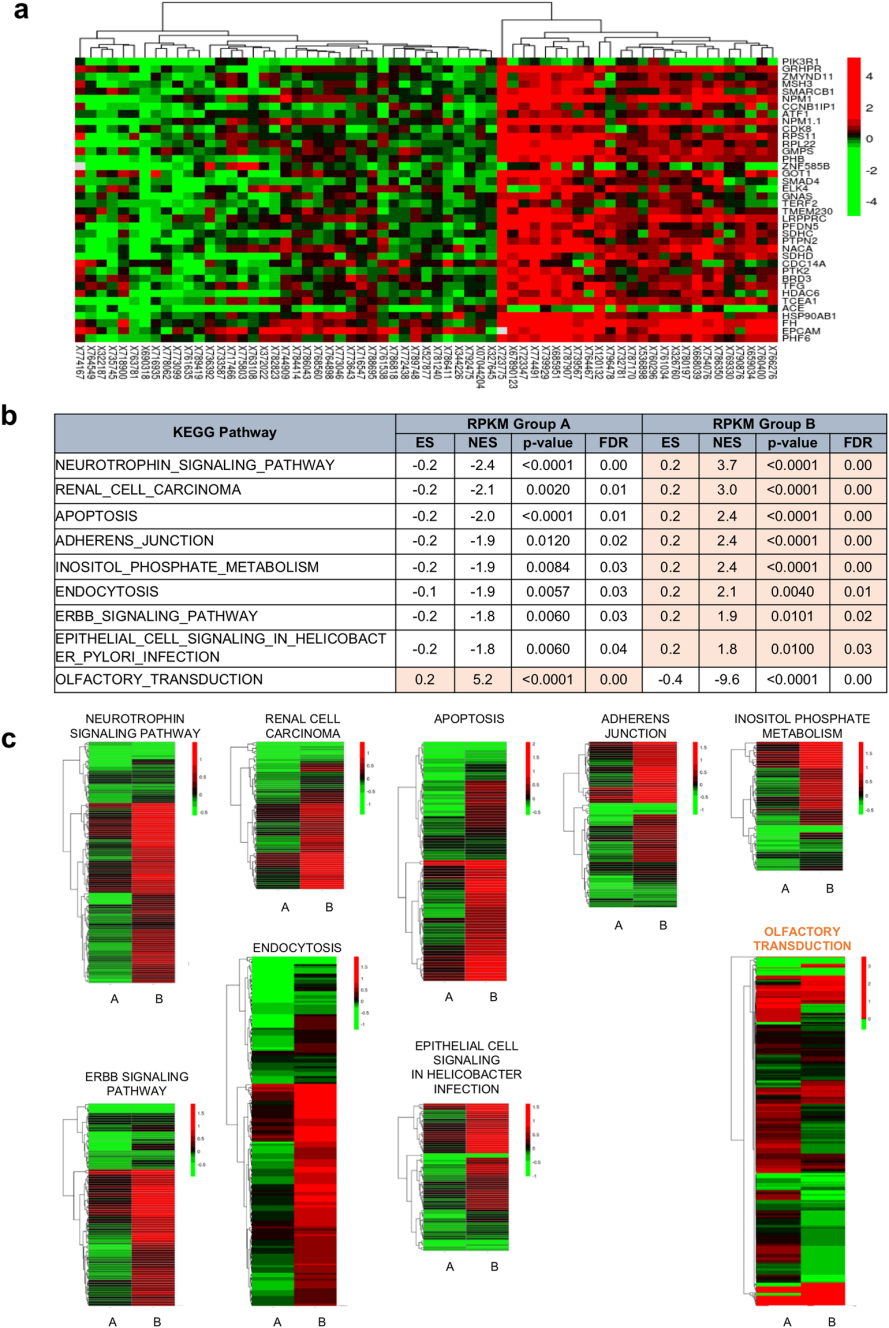
The urine 37 gene signature distinguishes PCa patients into two groups. (**a)** Unsupervised clustering of log-fold change (logFC) values shows that patients can be grouped into two groups, Group A and Group B, exhibiting lower and higher expression of the 37 genes compared to the matched normal, respectively. The logFC was obtained from the log2 ratio of RPKM values of tumor and matched normal of the listed genes. **(b,c)** KEGG pathway analysis suggests that the two molecularly identified groups are physiologically distinct, as shown by opposite enrichment of the depicted pathways. The logFC values were obtained by comparing tumor versus matched normal samples from each group via paired limma analysis.

Thus, we performed principal component analysis (PCA) of the 37 upregulated genes in the 65 patient tissue RNA-seq data. This 37-gene signature divided the tumor samples into two distinct groups, A and B (Fig. 3a), which did not differ with respect to Gleason score, tumor stage, or metastasis status (Supplementary Fig. 3). However, the two groups did show significant differences in *PCA3* and *KLK3* expression (Supplementary Fig. 4), which were both significantly higher in group B than in group A patients (Supplementary Fig. 4).

With respect to pathway differences between groups, nine pathways were significantly different: eight pathways were significantly upregulated (FDR > 0.05) in group B, and one pathway, olfactory transduction, was upregulated in group A (Fig. 3b,c). Therefore, the 37-gene signature in urine samples represents prostate tissue gene expression and might be useful to distinguish advanced PCa (higher *PCA3* and *KLK3* levels in cancer) as well as to detect PCa itself.

### Metabolomic profiling of urine from normal subjects and patients with diseased prostates

**Figure 4:**
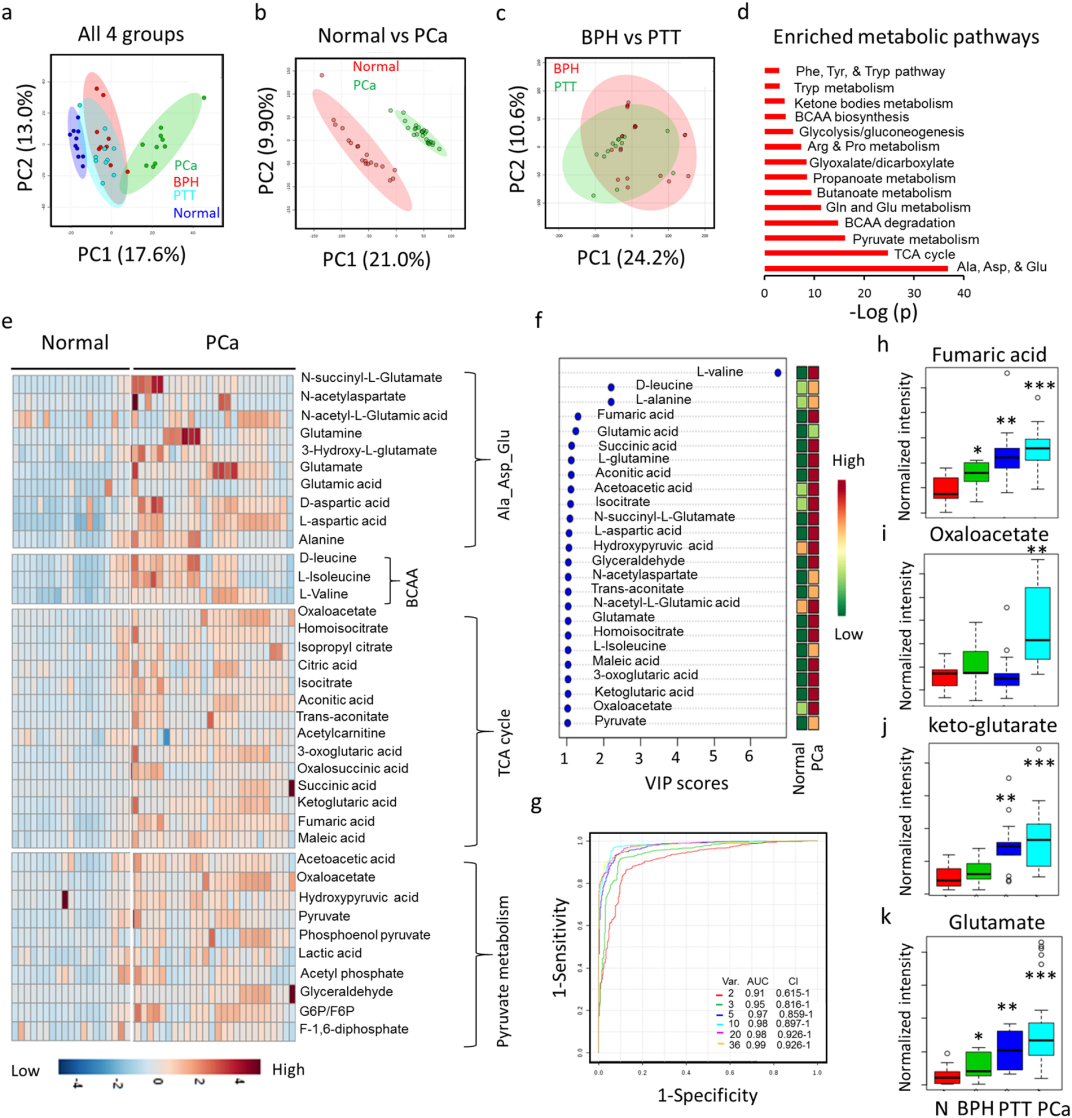
Global untargeted urine metabolomics profile data reveals distinct metabolic differences between PCa and normal. (**a)** Multivariate principal component analysis (PCA) scores plotted for normal (n = 20), BPH (n = 20), PTT (n = 11), and PCa (n = 20). **(b)** PCA score plot for BPH and PTT. **(c)** PCA score plot for normal and PCa. **(d)** List of significant metabolic pathways in PCa urine samples. The p-value is shown in negative log_10_ scale. **(e)** Heat map of the differential metabolites in PCa tissue. Color bar indicates the relative abundance of the metabolites, with red indicating a higher concentration and blue indicating a lower concentration. Ala_asp_Glu, alanine, aspartate, and glutamate metabolism; BCAA, branched chain amino acid metabolism, G6P, glucose-6-phosphate; F6P, fructose-6-phosphate. P < 0.05 was considered significant. (**f**) Metabolites ranked by their contributions and shown as variable importance in the projection (VIP) scores. (**g**) ROC curves for the predictive model. Shown as combination metabolite models calculated from the logistic regression analysis. (**h–k**) Significantly higher levels of metabolites in the TCA cycle (**h,i,j**) and alanine, aspartate, and glutamate metabolism (**k**) pathways were found in the PCa urine samples compare to normal, BPH, and PTT.

With this in mind, we performed global metabolite profiling of urine from patients with normal prostates, BPH, PTT, and PCa to discover cancer-specific metabolic changes. In global metabolite profiling, the metabolic profiles of urine specimens from normal subjects and patients with cancer were distinct and separate by PCA (Fig. 4a,b), whilst there was significant overlap between the profiles obtained from patients with BPH and PTT (Fig. 4c). Positive and negative ion data were first normalized to the specific gravity and then normalized to the total ion signal for all subsequent statistical analyses (Supplementary Fig. 5a). Positive and negative ion data sets were treated separately, and initial analysis was performed with PCA. The negative ion data set separation by PCA was very distinct between PCa and control groups, with BPH and PTT clustering together but as a separate cluster from PCa and control. Separation was primarily observed along PC1 (Fig. 4a). However, no correlation was observed between PSA scores and metabolic profiling. This result was reproducible with a second set of urine samples (Supplementary Fig. 5b). Further, subsequent hierarchical clustering-based heatmap analysis revealed distinctly higher abundance of global metabolites in PCa urine samples compared to normal samples (Supplementary Fig. 5c); over 180 metabolites in positive ion mode and 140 metabolites in negative ion mode were detected from the extracted urine samples, respectively.

Comprehensive metabolic networks in PCa urine samples have not been well studied, and understanding differential metabolic pathway utilization in PCa might contribute towards the development of robust biomarkers. Therefore, we performed metabolite-based pathway enrichment analysis (Supplementary Fig. 5d), which revealed 14 significantly impacted metabolic pathways in the urine metabolome (Fig. 4d). Notably, alanine, aspartate, and glutamate metabolism, TCA cycle metabolism, pyruvate metabolism, and several amino acid pathway metabolites were identified at higher levels in PCa urine samples compare to normal (Fig. 4e), consistent with previous tissue-based studies^16,25^.

We next conducted metabolite-based urinary biomarker screening, with metabolite contribution assessed by examining the variable importance in projection (VIP) score, which is calculated from the weighted sum of the square for each partial least square design (PLS) loadings for each principal component. Of the top twenty five variables identified by VIP scores, all were metabolite variables that significantly contributed to the class separation of normal and PCa samples (Fig. 4f). We then conducted multivariate receiver operating characteristics (ROC) curve-based exploratory biomarker analysis to identify a diagnostic PCa-related metabolite signature. To better predict PCa, the top 50 discriminatory metabolites were identified via logistic regression (Fig. 4g). A combination of six metabolites showed better discrimination (AUC > 98%) than each metabolite individually (AUC<91%) (Fig. 4g): aconitic acid (AUC = 0.97), succinic acid (0.96), fumaric acid (AUC = 0.955), oxaloacetate (AUC = 0.952), α-ketoglutaric acid (AUC = 0.921), and glutamate (AUC = 0.951) (Supplementary Fig. 6). Subsequent data analysis identified significantly higher levels of TCA cycle metabolites (fumaric acid, oxaloacetate, and keto-glutaric acid) (Fig. 4h–j) and glutamate metabolism (glutamate) pathway (Fig. 4k) metabolites in PCa urine samples compared to urine samples from healthy and inflammatory prostates such as BPH and PTT.

**Figure 5:**
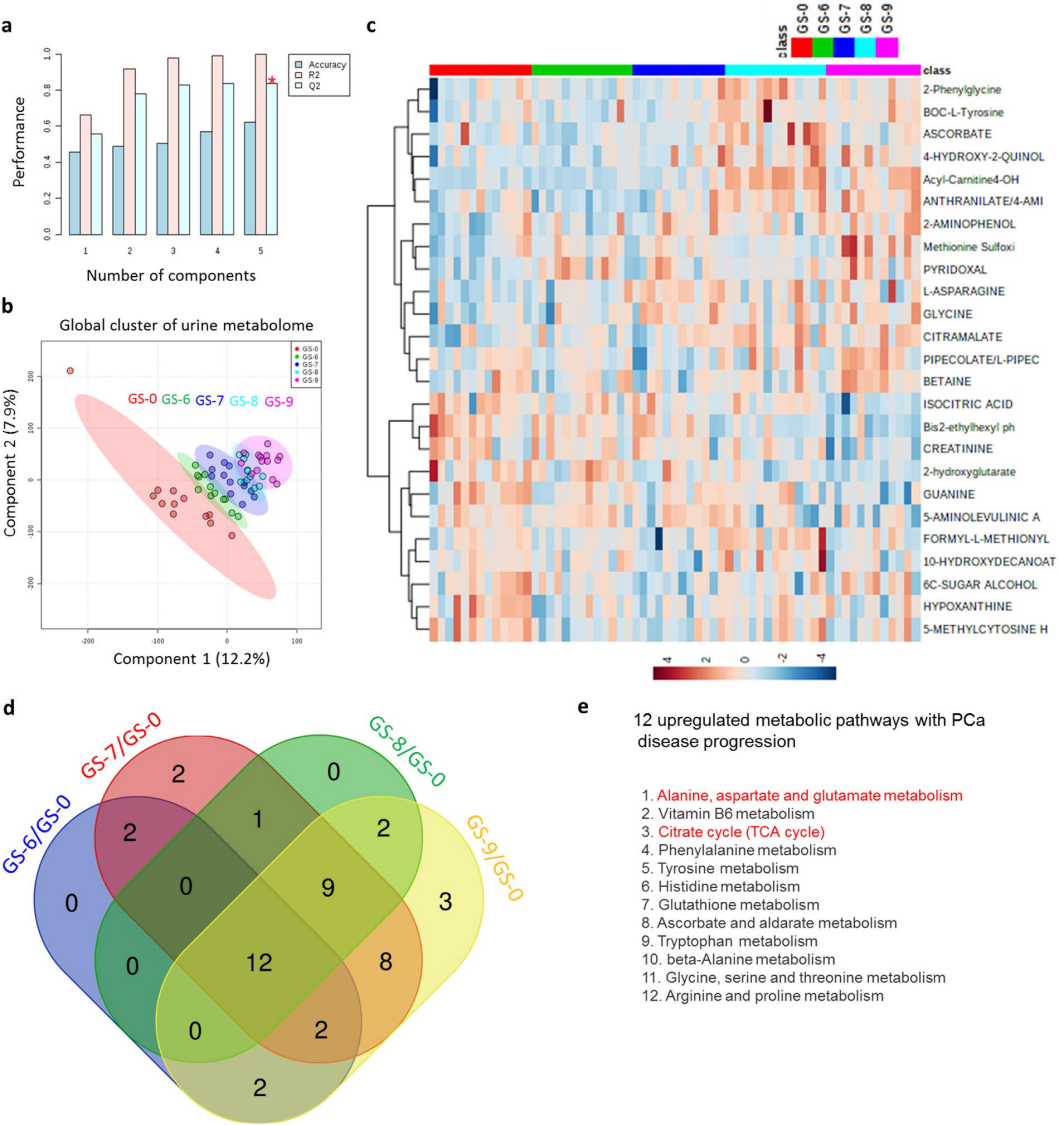
Correlation of TCA cycle and glutamate metabolism pathway metabolite levels with PCa Gleason score (GS). **(a)** Validation of PLS-DA (partial least square-differential analysis) model. Q2 indicates the accuracy of the model is over 80% when it includes the top five components. **(b)** Plot of PLS-DA component 1 and component 2 for log2 autoscaled metabolite abundance data for normal versus different PCa GS. Circles indicate the 95% confidence intervals of each sample group. **(c)** Heatmap showing the autoscaled abundances of the top 25 untargeted metabolites in normal versus different PCa GS-based urine samples. Data were analyzed using the Euclidean distance calculation and ward clustering algorithm. **(d,e)** Venn diagram represents common and unique metabolic pathways that were significantly upregulated in PCa disease progression. (**f**) List of the top 12 significant metabolic pathways that were commonly screened as upregulated in different GS of PCa.

Given that Gleason score (GS) status correlates with PCa tumor progression, an additional independent 11 normal, 11 GS-6, 11 GS-7, 11 GS-8, and 11 GS-9 PCa urine samples were collected and again subjected to global metabolomics analysis using mass spectrometry (Fig. 5). Metabolite data were analyzed using same statistical approaches as in Fig. 4. Furthermore, metabolome data accuracy was validated by a partial least squares-discriminant analysis (PLS-DA)-based Q2 model (Fig. 5a). Supervised multivariate statistical analysis of the global metabolome revealed a profound trend of clustering with respect to the four different GS groups and the normal urine samples (Fig. 5b). Hierarchical clustering heatmap analysis revealed the top 25 distinct metabolite signatures among normal and different GS groups (Fig. 5c). Metabolic pathway analysis between normal versus individual GS groups identified several potentially unique and common metabolic networks (Supplementary Fig. 7a–d). Differential metabolites from four GS groups and integration of their corresponding biochemical pathway analysis revealed twelve metabolic networks that were commonly present and upregulated with increased GS score (Fig. 5d). Among these pathways, TCA cycle and glutamate metabolism pathways were the pathways most upregulated with PCa disease progression (Fig. 5e). Notably, levels of TCA cycle and glutamate metabolism pathway metabolites were significantly increased with the highest GS, the most aggressive form of PCa (Supplementary Fig. 7e–j).

### Integrated gene expression and metabolite analysis

**Figure 6:**
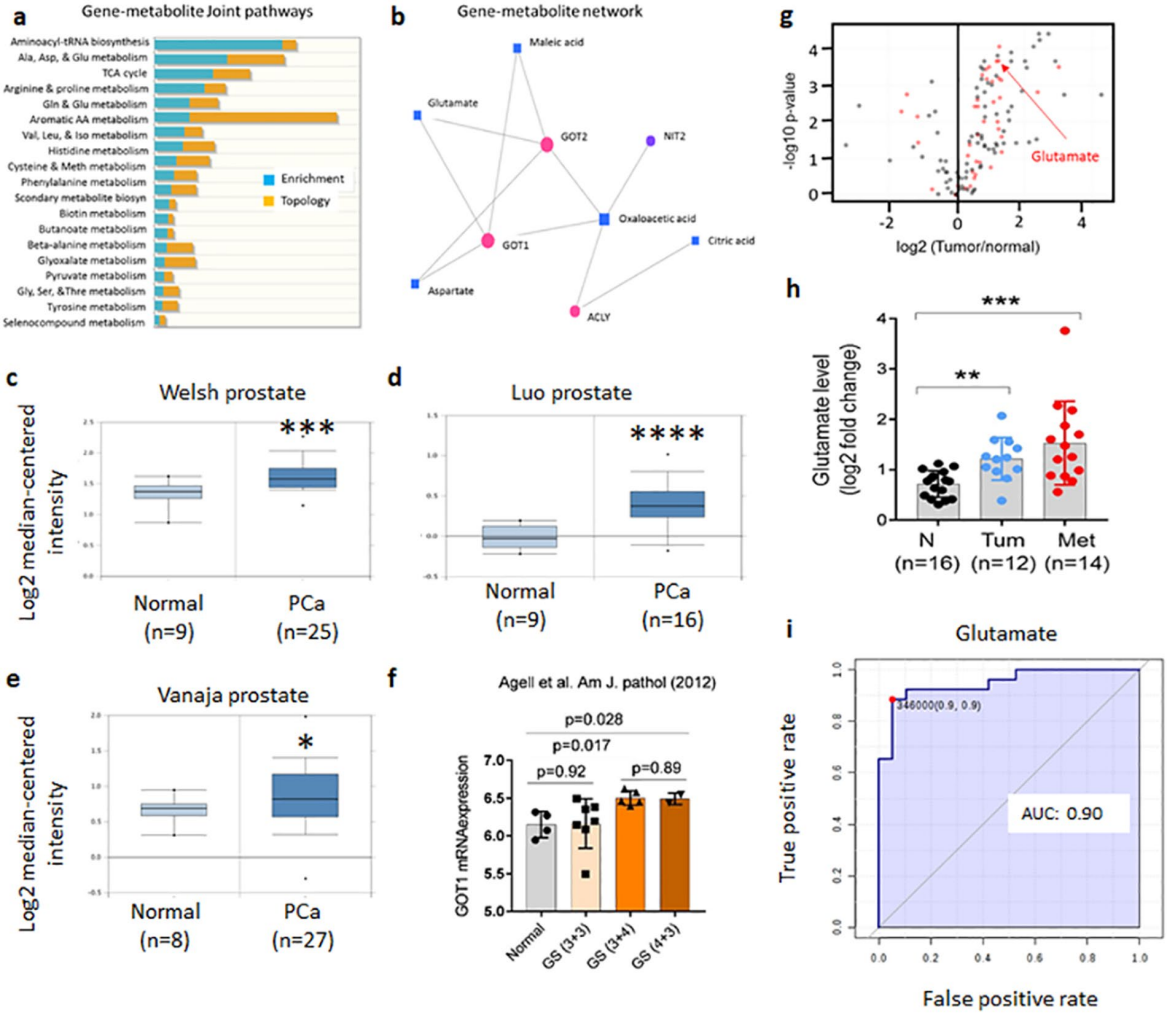
Integrated metabolic pathway enrichment analysis. (**a)** Gene-metabolite joint pathways were identified using the MetaboAnalyst integrated pathway analysis module. **(b)** Gene-metabolite network exploration identified a GOT1- and GOT2-mediated interactive network that might influence the TCA cycle and other metabolic pathways. **(c–e)** Box plots of GOT1 expression in malignant prostate tumor compared to normal (data sources https://www.oncomine.org/resource/login.html). **(f)** A bar graph of GOT1 expression across the prostate cancer Gleason score (GS) (data sources GSE30521). **(g–i**) A volcano plots of the differential metabolites **(g)**, bar graph of glutamate level among normal, tumor, and metastatic clinical specimens of Pan-Cancer Metabolism Data Explorer **(h**), and ROC curve for glutamate level prediction in PCa urine samples compared to normal **(i**). Statistical significance was analyzed by Student’s t-test.

We reasoned that integrating changes in gene expression and metabolite levels evident in the urine samples would better reveal the key pathways driving PCa and hence pinpoint the most robust biomarkers. The integrated pathway analysis module of MetaboAnalyst^26^ was used to map both genes and metabolites to KEGG pathways to determine not just overrepresented pathways but also the relative importance of the genes and compounds based on their relative locations (topology). The top three pathways most significantly enriched for differentially expressed genes and metabolites were: aminoacyl-tRNA biosynthesis; Ala, Asp, and Glu metabolism; and the TCA cycle) (p < 0.001; Fig. 6a and Supplementary Table 6). Aminoacyl-tRNA biosynthesis probably represents an increase in global protein translation and demand for protein synthesis in cancer cells^27^. However, Ala, Asp, and Glu metabolism and the TCA cycle are closely related pathways that are critical for energy generation and carbon and nitrogen metabolism for biomass accumulation^28^, especially in rapidly dividing cells such as cancer cells.

Analysis of the top 25 significant metabolites identified by UHPLC-HRMS and their corresponding genes from RNA-seq revealed that GOT1- and GOT2-mediated metabolism was the main gene-metabolite interactive node influencing Ala, Asp, and Glu metabolism and the TCA cycle metabolism (Fig. 6b). In several PCa clinical datasets, GOT1 expression was significantly higher in malignant prostate compared to normal (Fig. 6c–e), whereas GOT2 expression showed no significant differences (data not shown).

Interestingly, *GOT1* expression was significantly elevated in high GS tumors compared to controls (Fig. 6f). *GOT1* expression was significantly elevated in GS 7 tumors [both (3 + 4) and (4 + 3)] compared to normal. Furthermore, we identified aberrant levels of glutamate, the product of GOT1, in primary and metastatic prostate cancers compared to normal (Fig. 6g,h). We also evaluated the ROC curves for glutamate levels in PCa urine specimens normalized to BPH/PTT, and glutamate had an AUC of 90% (Fig. 6i). Therefore, GOT1-mediated glutamate metabolism might be critical for PCa disease progression, and a better understanding of GOT1-driven metabolism could reveal potential drug targets and biomarkers for PCa.

### Glutamate metabolism contributes to the cancerous phenotype via *GOT1*-mediated redox balance

**Figure 7:**
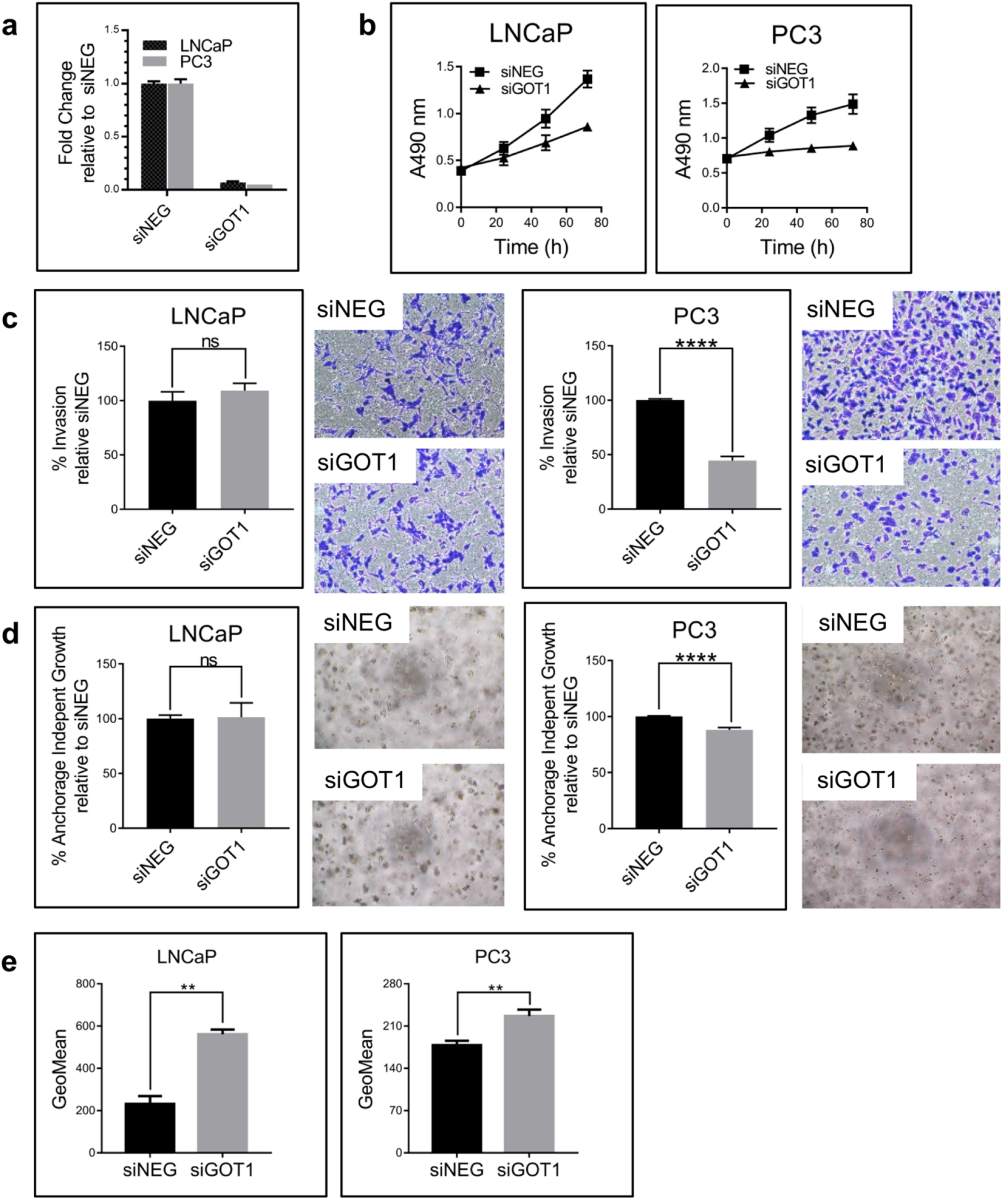
GOT1 supports proliferation, invasion, and colony formation in prostate cancer cell lines. (**a)** GOT1 knockdown in LNCaP and PC3 prostate cancer cell lines. **(b)** GOT1 knockdown significantly inhibits cell viability in PC3 and LNCaP cells. **(c)** Invasion and **(d)** anchorage-independent growth in prostate cancer cell lines upon GOT1 knockdown. **(e)** GOT1 knockdown significantly increases ROS production in PC3 and LNCaP cells. The data from three independent experiments were expressed as mean ± SD.

*GOT1*, a cytosolic transaminase that converts Asp to Glu, and other genes involved in glutamine metabolism such as *GLUD1*, *GOT1*, *GOT2*, and *MDH1* were significantly upregulated in PCa urine samples (Fig. 2b and Supplementary Table 7). To investigate GOT1’s role as a regulatory metabolic node in prostate cancer, we knocked down *GOT1* in the prostate cancer cell lines LNCaP and PC3 using siRNA (Fig. 7a). As expected, *GOT1* knockdown upregulated the upstream metabolites (Supplementary Fig. 8) Glu [1.2-fold (LNCaP; *p* = *0.01*) and 1.4-fold (PC3; *p* = *0.03*)] and Asp [1.5-fold (LNCaP; *p* = *0.0004*) and 2.6-fold (PC3; *p* = *0.0006*)] in both cell lines. *GOT1* knockdown significantly decreased the viability of both LNCaP and PC3 cells (Fig. 7b), consistent with previous reports that GOT1 repression suppresses tumor growth^29,30^, and the invasiveness and colony-forming ability of PC3 cells (Fig. 7c,d). We therefore examined the mechanism by which *GOT1* regulated prostate cancer cell viability.

Maintaining NAD/NADH balance supports *de novo* aspartic acid (Asp) biosynthesis and is required for proliferation^31,32^. Since GOT1 is part of the malate-Asp shuttle^31^, we checked whether *GOT1* knockdown affected the NAD/NADH ratio; the NAD/NADH ratio was indeed decreased (Supplementary Fig. 9), suggesting that this reduction may have influenced the cell proliferation inhibition in both LNCaP and PC3 cells. As depicted in Fig. 2b, GOT1 is necessary to convert Asp derived from the Gln TCA cycle into oxaloacetate and malate to produce NADPH^33^, which is essential for maintaining intracellular redox balance via detoxification of damaging reactive oxygen species (ROS). Both LNCaP and PC3 cells showed increased ROS levels upon *GOT1* knockdown (Fig. 7e), suggesting that GOT1 plays a role in cellular redox balance and can be manipulated to reduce the viability of prostate cancer cells.

## Discussion

Recent advances in transcriptomics and metabolomics have led to the identification of various candidate biomarkers for cancer diagnosis and prognosis^16,34^. However, biomarkers derived from one dataset may not be reliable, and reproducibility in independent cohorts is challenging^35^.

Prostate carcinogenesis is known to involve metabolic reprogramming to provide sufficient energy for rapid cellular proliferation^28,36^. Many cancer cells exhibit augmented aerobic glycolysis, that previously reported as the Warburg effect, even in high-oxygen environments^37^. This metabolic adaptation helps to provide essential cellular components such as lipids and nucleotides to support the anabolic needs of rapidly proliferating tumor cells. Beyond the Warburg effect, the TCA cycle and oxidative phosphorylation are known to play important roles in PCa^36,38^. Prostate epithelial cells normally produce certain components of prostatic fluid such as citrate, PSA, and polyamines^38^. Increased citrate production by prostate cells means that they favor citrate synthesis over citrate utilization. However, PCa cells degrade citrate and accumulate oxidized citrate, resulting in more efficient energy production^36,39^.

There are significant advantages to using biofluids including blood, urine, saliva, and seminal plasma as sources of biomarkers^40,41^. Among them, urine is a promising liquid biopsy as it is noninvasive, replenishable, and convenient to collect. Urine has been at the center of clinical proteomics and provided biomarkers for renal disease^42^, renal cell carcinoma^43^, bladder cancer^44^, and prostate cancer^45^. In addition to proteins and peptides, urine contains various nucleic acids, metabolites, and lipids. Recently, the long noncoding RNA *PCA3* and the fusion gene *TMPRSS2:ERG* have been proposed as urinary PCa biomarkers^46^. Here we report for the first time a global transcriptomic profile of PCa in urine. We applied capture-based enrichment (RNA Access protocol), in which probes target exonic regions, and were able to separate PCa samples from normal healthy individual samples by unsupervised methods (Supplementary Fig. 2). Targeted or global strategies have been used to profile metabolites in urine samples and identify PCa biomarkers^14,16,47,48^, but results have been highly variable^49^. In the first unbiased metabolomics study measuring 1126 metabolites in 262 clinical samples including 110 urine samples, the glycine derivative sarcosine was elevated in PCa tissue and urine from PCa patients, and functional validation of the oncogenic role of sarcosine was provided *in vitro*^16^. However, sarcosine was not a reproducible prognostic marker in independent cohorts^15,17^, a common finding in single-biomarker studies that possess neither the specificity nor sensitivity for clinical development^50^.

We have shown that urine global metabolite profiling could distinguishe PCa patient samples from normal prostates, BPH, and PTT (Fig. 4). Furthermore, global metabolomics analysis was able to distinguish the four different GS groups and the normal urine samples (Fig. 5). Recently, due to the limitations of Gleason grading, a new PCa grading system proposed by Epstein and colleagues was introduced, which assigns the tumor histologic patterns 1 through 5^51^. Notably, the new system distinguishes Gleason score 3 + 4 = 7 (Group 2) and 4 + 3 = 7 (Group 3), which are prognostically very different. Due to the limited patient information available, we were not able to determine the metabolic profile differences between Groups 2 and 3; it would be interesting to analyze the metabolic profiles of these two groups in the future to establish whether they are distinguishable. The recently proposed prostate cancer grading system also divides GS 8–10 disease into GS 8 and GS 9–10 on the foundation of biochemical repetition (BCR) succeeding radical prostatectomy (RP) as an outcome. However, BCR does not inevitably portend worse survival outcomes, so this is a less important distinction to make biochemically.

In pancreatic ductal adenocarcinoma (PDAC), GOT1 is required to sustain cell growth by enabling the production of NADPH to compensate for internal ROS^33^. We showed that GOT1 is essential for PCa cell line (PC3 and LNCaP) growth. *GOT1* knockdown increased ROS levels, suggesting that GOT1 may be involved in NADPH generation. Consistent with this, chronic exposure to cycling anoxia/re-oxygenation induced GOT1 in DU145 prostate cancer cells, that decreased cellular ROS levels with increased glutathione^52^. It would be interesting to establish if *GOT1* knocked-down PC3 and LNCaP cells are more sensitive to ROS-inducing treatments such as ionizing radiation. In PDAC, GOT1 plays a role in anaplerotic metabolism countering ROS production in low pH conditions^53^. However, this is unlikely to be the case in PCa. Pancreatic cancer cell growth was reduced in low pH conditions, but acidic pH stimulated both proliferation and colony formation of PC3 cells^54^, while LNCaP cells underwent apoptosis^55^. Although the effect of low pH in PCa cells was not similar to PDAC cells, it remains to be determined whether low pH increases GOT1 levels or whether anaplerotic glutamine metabolism is affected by pH in PCa cells.

As reported previously, GOT1 also functions as a member of the malate-aspartate shuttle^32^, in which two pairs of enzymes, glutamate oxaloacetate transaminases (GOT) and malate dehydrogenase (MDH), serve to transfer reducing equivalents across the mitochondrial membrane (Fig. 2b). Our transcriptomic analysis revealed the upregulation of all members of the shuttle including *GOT1*, *GOT2*, *MDH1*, and *MDH2* (Supplementary Table 7). These results suggest that the malate-aspartate shuttle may play an important role in cell growth in PCa. This hypothesis is supported by the reduction in NAD/NADH ratio upon *GOT1* knockdown in both cell lines. In agreement with our observation, Zhou *et al*. showed that GOT1 inhibition in osteosarcoma and lung cancer cell lines decreased proliferation and NADH accumulation. Higher GOT1 expression was linked to poor survival in thyroid carcinoma, breast carcinoma, and lung adenocarcinoma, suggesting that GOT1 also plays a pivotal role in other cancer types^56^.

## Conclusions

In this study we identified a group of RNA and metabolite signatures in urine as putative PCa biomarkers and therapeutic targets. To improve the accuracy of disease classification, we carried out metabolic and transcriptomic profiling of urine obtained from BPH, PTT, and PCa patients (without prostatic massage). Urine from normal healthy individuals was used as the control. Through an integrated analysis of metabolomic and transcriptomic data, we identified GOT1 as a key regulator of metabolic changes in PCa patients. Prostate cancers appear to undergo GOT1-dependent metabolic adaptation to promote a malignant phenotype and resist oxidative stress. The glutamate phenotype represented by the gene expression and metabolic changes in urine reflect this GOT1-dependent pathway in PCa cells. In addition to focusing on these pathway components as urine biomarkers of PCa, enzymes involved in this pathway might be excellent targets for PCa therapy. Indeed, small molecule inhibitors of GLS1 (mitochondrial glutaminase), which converts glutamine to glutamate, already exist^57,58^. Targeting this pathway is worthy of further investigation either with or without concurrent ROS-induced cellular stress^57^, this latter approach a particularly appealing strategy in patients with prostate cancers treated with ionizing radiotherapy. Finally, we postulate that liquid biopsies are an extremely useful tool for non-invasive biomarker and target discovery not only in PCa but also in other cancers.

## Methods

### Sample collection and preparation

The Sanford Burnham Prebys Medical Discovery Institute, Adventishealth (formerly named as the Florida Hospital) and the John Hopkins University School of Medicine IRB protocols approved the study to consent participants and collect samples (patient and healthy individual urine and tissue samples). We have obtained the consent from all subjects, and the samples were de-identified prior distributing to the lab members. Our studies agreed to the principles set out in the WMA Declaration of Helsinki and the Department of Health Human Services Belmont Report.

This was a retrospective study of individuals attending the Global Robotics Institute (Celebration, FL, USA) and Florida Urology Associates (Orlando, FL, USA) of Florida Hospital and the Johns Hopkins Hospital between 2008 and 2014. Urine samples were collected from 20 benign prostatic hyperplasia (BPH), 11 prostatitis (PTT), and 20 prostate cancer (PCa) patients and 20 normal healthy individuals with no history of cancer attending the Global Robotics Institute and Florida Urology Associates of Florida Hospital. An additional 55 urine samples (11 samples from each different Gleason score) were obtained from patients attending the Johns Hopkins Hospital. Urine samples were collected using urine preservation tubes (Norgen Bioteck, Thorold, ON, Canada) and kept at room temperature until centrifugation to separate the exfoliated cells in the urine samples. Cell-free urine was then stored at −80 °C until further use for metabolite analysis. The exfoliated cells from normal and PCa urine samples were used for total RNA purification using the Urine (Exfoliated Cell) RNA purification kit (Norgen Bioteck). Total RNA was subjected to RNA-seq to identify gene signatures.

### Global untargeted metabolomics

We conducted global metabolomics using ultra-high performance liquid chromatography coupled with high-resolution mass spectrometry (UHPLC-HRMS) on a Thermo Q Exactive with Dionex UHPLC (Thermo Fisher Scientific, Waltham, MA). To 50 µL of urine, 20 µL of internal standard was added (40 µg/mL tryptophan-d3, 4 µg/mL leucine-d10, 4 µg/mL creatine-d3, and 4 µg/mL caffeine-d3) followed by 400 µL of 98:2 acetonitrile:water with 0.1% sodium azide. The solution was vortexed and spun down at 20,000 x g (8 °C) for 10 min. Transferred supernatant was dried under a gentle stream of nitrogen and the dried sample restored in 50 µL of 0.1% formic acid in water was transferred to a LC vial with a fused glass insert for analysis. Liquid chromatography–high resolution mass spectrometry (LC-HRMS)^59,60^ analysis was done in positive and negative ion modes as separate injections, injecting 2 µL for positive and 4 µL for negative ions. Separation was achieved on a C18-pfp column (ACE Excel 100×2.1 mm, 2 µm, Advanced Chromatography Technologies, Aberdeen, Scotland) with 0.1% formic acid in water as A and acetonitrile as B. An in-house retention library of 600 metabolites was used to identify metabolites. All the data normalization, multivariate analyses, pathway analysis, and biomarker discovery were carried out using Metaboanalyst 4.0 (http://www.metaboanalyst.ca). Integrated gene-metabolite network analysis was conducted using the Metascape plugin (http://metscape.ncibi.org/) in the Cytoscape (http://www.cytoscape.org/).

### Cell culture

PCa cell lines, PC3 (ATCC® CRL-7934™) and LNCaP (ATCC® CRL-1740™) were cultured in Dulbecco’s Modified Eagle Medium and RPMI 1640 medium (Thermo Fisher Scientific), respectively, with 10% FBS and penicillin/streptomycin.

### Quantitative real-time PCR (qPCR)

Total RNAs from cell lines were purified using the Direct-zol RNA Miniprep kit (Zymo Research, Irvine, CA). Normal prostate epithelial cell RNA was purchased from BioChain Institute Inc. (Catalog # R1234201-50, Newark, CA). RNA (0.5 µg) was then used for cDNA synthesis using a high capacity cDNA reverse transcription kit (Applied Biosystems, Foster City, CA). qPCR was performed using a Power SYBR Green PCR master mix (Applied Biosystems) in the 7500 Real-Time PCR system (Applied Biosystems). A final reaction volume of 10 µl was used containing 1 µl (corresponding to 10 ng) of cDNA template, 5 µl of 2X Power SYBR Green PCR master mix (Applied Biosystems), and 0.2 µM of each primer. The reaction was subjected to denaturation at 95 °C for 10 min followed by 40 cycles of denaturation at 95 °C for 15 sec and annealing at 58 °C for 1 min. The comparative Ct analysis was performed using SDS1.2.3 software (Applied Biosystems) with TATA-box binding protein (TBP), β-actin (ACTB), and hypoxanthine phosphoribosyltransferase 1 (HPRT1) serving as endogenous controls (average Ct value of three control genes was used for calculating ΔCt value). The primer sequences for the genes are listed in Supplementary Table 8.

### RNA access

The quantity and integrity of the RNA was measured using both the Qubit RNA HS Assay Kit (Thermo Fisher Scientific) and the Agilent 2100 Bioanalyzer RNA Pico kit (Agilent Technologies, Santa Clara, CA). Following the Illumina DV_200_ metric (percentage of RNA fragments greater than 200 nucleotides), 100 ng of RNA with DV_200_ > 30% was used to prepare sequencing libraries in accordance with the TruSeq RNA Access protocol (Illumina, Inc., San Diego, CA). The random primers were used for the first strand cDNA synthesis followed by second strand synthesis. The cDNA then underwent 3′ adenylation followed by adapter ligation and PCR amplification (15 cycles). Library quality was measured using both the Qubit dsDNA HS Assay Kit and Agilent Bioanalyzer DNA kit. A 4-plex pool of libraries was then made (200 ng of each sample) followed by two rounds of hybridization/capture and a final amplification (10 cycles). The quality and quantity of the final libraries were determined using the Agilent 2100 Bioanalyzer DNA HS kit and Kapa Biosystems qPCR (Kapa Biosystems, Inc., Wilmington, MA). Multiplexed libraries were pooled and normalized to 17.5 pM. The libraries were sequenced using a 75 bp paired-end run on the Illumina MiSeq instrument. Reads were mapped to the hg19 human genome using tophat2.0.1; mapped reads were filtered based on the mapping quality. The overall mapping rates were about 93%. mRNA quantification was conducted in Partek Genomics Suite 6.6. The differential expression of mRNAs was analyzed using a R package edgeR.

### Clustering and principal component analysis (PCA)

The resulting mRNA expression profile included 46,459 transcripts, and the non-parametric Mann-Whitney U-test was used to identify significantly regulated transcripts. 5510 transcripts were identified (p ≤ 0.05) as significantly differentially expressed between normal and PCa groups. Within those 5,510 transcripts, 1,118 transcripts had Reads Per Kilobase of transcript per Million mapped reads (RPKM) values greater than 1.0 for all samples. We pre-compiled a gene panel that listed all prostate cancer-related genes (with the help of Illumina). By comparing with this panel, we obtained 542 transcripts with RPKMs greater than 1.0 for all samples. Within these 542 transcripts, 116 were significantly regulated (Table 1). All overlapping transcripts between the expression profile and prostate cancer panel, a total of 3825 transcripts, were used to run the unsupervised clustering analysis and PCA. Hierarchical cluster analysis was performed in R using the correlation between samples to characterize similarity. Each sample was assigned to its own group and cluster algorithm was repeated until there was a single cluster. Correlations between samples were calculated using the expression values of the 3825 transcripts. We also used PCA to visualize sample-to-sample distance. The transformation was defined that the first principal component accounted for the largest variance (as much of the variability in the dataset as possible). In the results, each sample was projected onto the 3D space in which the three axes were the first three highest principle components (see Supplementary Fig. 2b).

### Gene set enrichment analysis (GSEA) of tissue RNA-seq data

Gene expression was analyzed in 65 patients using RNA-seq data^25^. Briefly, RPKM values from tumor and matched normal samples from 65 patients were analyzed for differential gene expression between tumor and matched normal using the Bioconductor limma package^61^. To compare between groups for tumor or matched normal tissues, linear model analysis using the limma package was used on RPKM values for all genes. The analysis was performed in R (version 3.4.4, www.R-project.org).

GSEA for Kyoto Encyclopedia of Genes and Genomes (KEGG) pathways was conducted using the GSEA desktop application (borad.mit.edu/gsea). All genes were ranked using scores based on fold-change direction and p-value, and enrichment analysis was conducted using GSEA pre-ranked with the ‘classic’ enrichment statistic. Significantly enriched genes sets were identified using q-value ≤0.05 as a cutoff.

### Transient transfection and cell proliferation assay

Cells (0.3 × 10^6^ cells) were mixed with siRNA (Thermo Fisher Scientific; final concentration 20 nM) and lipofectamine RNAiMAX (Thermo Fisher Scientific) mixture in 2 ml medium containing 10% FBS. In a 96-well plate, cells were seeded in duplicate at 7500 cells per wells and, after 24 h, the medium was replaced. Cell proliferation assay was performed using the CellTiter96 Aqueous One Solution Cell Proliferation Assay (MTS) kit (Promega, Madison, WI).

### NAD and NADH quantification

NAD/NADH ratio was determined using the NAD/NADH-Glo Assay kit (Promega). In a 96-well plate, cells were seeded in duplicate at 7500 cells per wells. Cells were lysed with base solution (100 mM sodium carbonate, 20 mM sodium bicarbonate, 10 mM nicotinamide, and 0.05% Triton X-100) with 1% dodecyltrimethylammonium bromide (Sigma Aldrich, St Louis, MO; D8638). Lysed cells were heated at 60 °C for 20 min in the presence/absence of acid, then subjected to the luciferase assay according to the manufacturer’s protocol.

### Measurement of reactive oxygen species (ROS)

One million cells were suspended in 1 ml medium having 20 μM 2′7′-dichlorofluoresicin diacetate (H_2_DCF-DA, Sigma Aldrich, D6883) and incubated for 30 min at 37 °C in 5% CO_2_. DCF fluorescence were detected using a FACSCalibur flow cytometer (Becton Dickinson, Anaheim, CA). Flowing Software v. 2.5.1 (www.flowingsoftware.com) was used for data analysis.

### Invasion assay

The cell invasion assay was performed using Corning BioCoat Matrigel Invasion Chambers (Discovery Labware, Bedford, MA). Cells were incubated in serum-free medium for 24 h and added to the upper chambers in serum-free medium (0.2 × 10^6^ cells). The lower chamber was filled with medium containing 10% fetal bovine serum. After 48 hr incubation at 37 °C, cells at the bottom side of upper chamber were stained with 0.5% crystal violet dye. The dye from invaded cells was extracted using methanol and absorbance at 570 nm was measured.

### Soft agar colony formation assay

Anchorage-independent growth was assessed using a CytoSelect 96-well cell transformation assay kit (Cell Biolabs Inc., San Diego, CA). Briefly, cells were seeded in soft agar at a density of 10,000 cells per well and incubated at 37 °C in 5% CO_2_ for 7 days. Colony formation was quantified with the 3-(4,5-dimethylthiazol-2-yl)-2,5-diphenyltetrazolium bromide (MTT) assay provided with the kit according to the assay protocol.

### Ethics approval and consent to participate

The institutional review board (IRB) of the Florida Hospital and The Johns Hopkins Hospital approved sample use. All participants were required to sign and provide written consent. All animal studies was conducted with the approval of the appropriate ethics committee in conformity with the ethical standards.

## Supplementary information

Supplementary information.

## Data Availability

The data are available from the corresponding author upon request.
